# Animal models of primary biliary cholangitis: status and challenges

**DOI:** 10.1186/s13578-023-01170-9

**Published:** 2023-11-22

**Authors:** Xu Wang, Yi Wei, Yanlei Yang, Yunjiao Yang, Haolong Li, Yongzhe Li, Fengchun Zhang, Li Wang

**Affiliations:** 1 Department of Rheumatology and Clinical Immunology, Peking Union Medical College Hospital, Peking Union Medical College, Chinese Academy of Medical Sciences, Key Laboratory of Rheumatology and Clinical Immunology, Ministry of Education, National Clinical Research Center for Dermatologic and Immunologic Diseases, Beijing, China; 2 Clinical Biobank, Department Medical Research Central, Peking Union Medical College Hospital, Chinese Academy of Medical Sciences and Peking Union Medical College, Beijing, China; 3 Department of Clinical Laboratory, Peking Union Medical College Hospital, Chinese Academy of Medical Science and Peking Union Medical College, Beijing, China

**Keywords:** Primary biliary cholangitis, Genetically modified models, Chemically inducible models, Biologically inducible models, Challenges

## Abstract

**Background:**

Primary biliary cholangitis (PBC) is an autoimmune liver disease. The aetiology of PBC remains unclear, and its pathogenesis is complex. Animal models are essential to clarify the pathogenesis of PBC and explore the occurrence of early events.

**Main body:**

Herein, we review recent research progress in PBC animal models, including genetically modified, chemically inducible, biologically inducible, and protein-immunised models. Although these animal models exhibit several immunological and pathological features of PBC, they all have limitations that constrain further research and weaken their connection with clinical practice.

**Conclusion:**

The review will benefit efforts to understand and optimise animal models in order to further clarify PBC pathogenesis and molecular targets for therapeutic interventions.

## Background

Primary biliary cholangitis (PBC), known as primary biliary cirrhosis until 2016, is an autoimmune liver disease [1]. The prevalence of PBC is increasing worldwide. China has the second-highest incidence rate of PBC in the Asia–Pacific region [2, 3], where the primary patients are 40–60-years-old women [2, 4]. Genetic susceptibility and environmental factors result in the loss of tolerance to self-antigens and multilineage immune dysregulation, inducing targeted damage in bile duct epithelial cells (BECs) [5, 6]. Multiple immune cells are involved in the pathogenesis of PBC. CD8^+^T cells target and destroy BECs, while CD4^+^T cells induce an inflammatory microenvironment around BECs by producing multiple cytokines, which recruit CD8^+^T cells. Further, in the early phase of the disease, Th1 cells are predominantly pro-inflammatory, and Th1 responses have been shown to predominate over Th2 responses in PBC. Th1 cytokines, such as interleukin(IL)-12, tumour necrosis factor(TNF)-α, and interferon (IFN)-γ, play an important role in the initiation and development of the disease. IL-12A and IL-12RB2 gene variants are strongly associated with PBC. Th17 cells are predominantly pro-fibrotic in the later phase of the disease. Th17 cells have the ability to produce high levels of IL-17, which accumulates around BECs. Moreover, IL-17 receptor and IL-23 receptor expression is upregulated in BECs [7]. Regulatory T cells (Tregs) maintain immune tolerance, but their numbers and inhibitory functions are down-regulated in PBC. Natural killer (NK) cells, NKT cells, dendritic cells, macrophages, and mucosal-associated invariant T cells are also important in the pathogenesis of PBC [8, 9]. Additionally, B cells produce antimitochondrial antibody (AMA) and infiltrate around BECs [10]. Elevated titres of AMA, which mainly targets the inner lipoyl domain within the E2 subunit of pyruvate dehydrogenase complex (PDC-E2), can be detected in 90–95% of patients [11]. Patients may also present with elevated alkaline phosphatase(ALP), Gamma-Glutamyl Transferase(GGT) and immunoglobulin(Ig)M. Histologically, PBC is characterised by intrahepatic BEC destruction and a dense lymphocytic infiltrate in the portal area. Granuloma formation and eosinophilic infiltration may occur early in PBC, which gradually progresses to liver fibrosis and cirrhosis in advanced stages [12]. Current therapies target the regulation of bile acid secretion [13]. However, whether BEC damage is the cause or result of autoimmune dysfunction remains unclear [14].

**Fig. 1:**
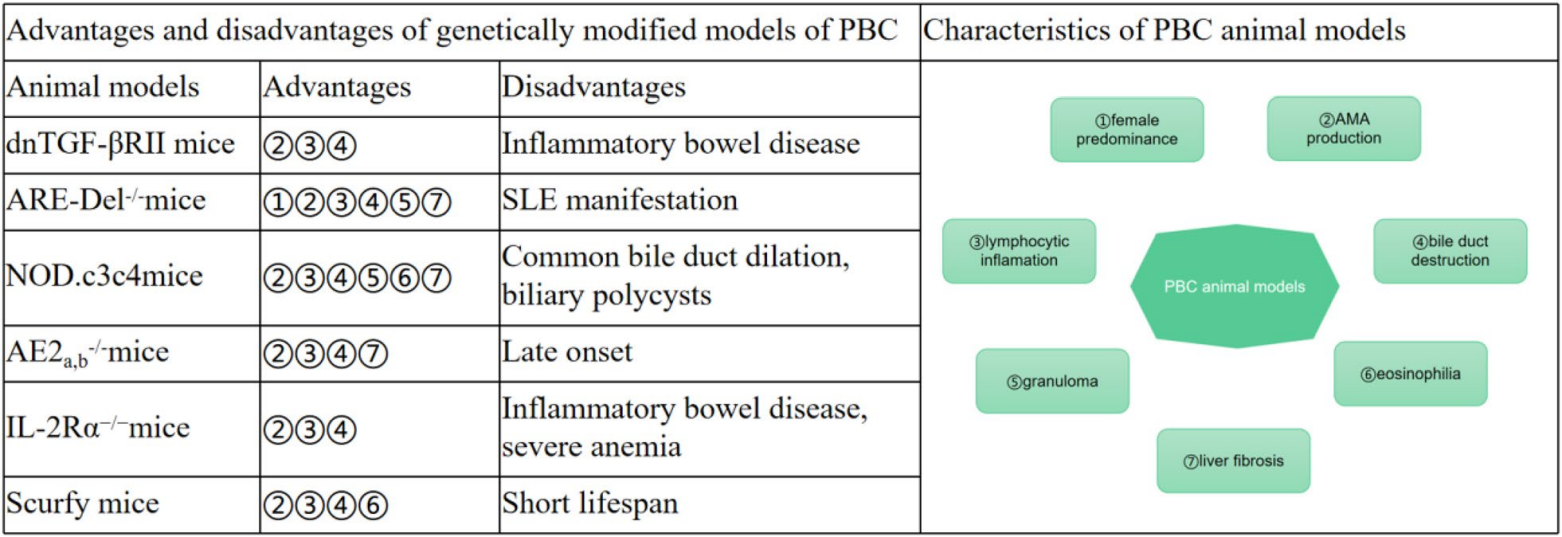
Advantages and disadvantages of genetically modified models of primary biliary cholangitis (PBC). Six genetically modified mouse models are available (dnTGF-βRII, IL-2Rα^−/−^, NOD.c3c4, AE2_a,b_^−/−^, ARE-Del^−/−^, and Scurfy mice). Each animal model has advantages and disadvantages (left). Animal models of PBC have the following characteristics: female predominance, antimitochondrial antibody production, lymphocytic inflammation, bile duct destruction, granuloma formation, eosinophilia, and liver fibrosis (right)

**Fig. 2:**
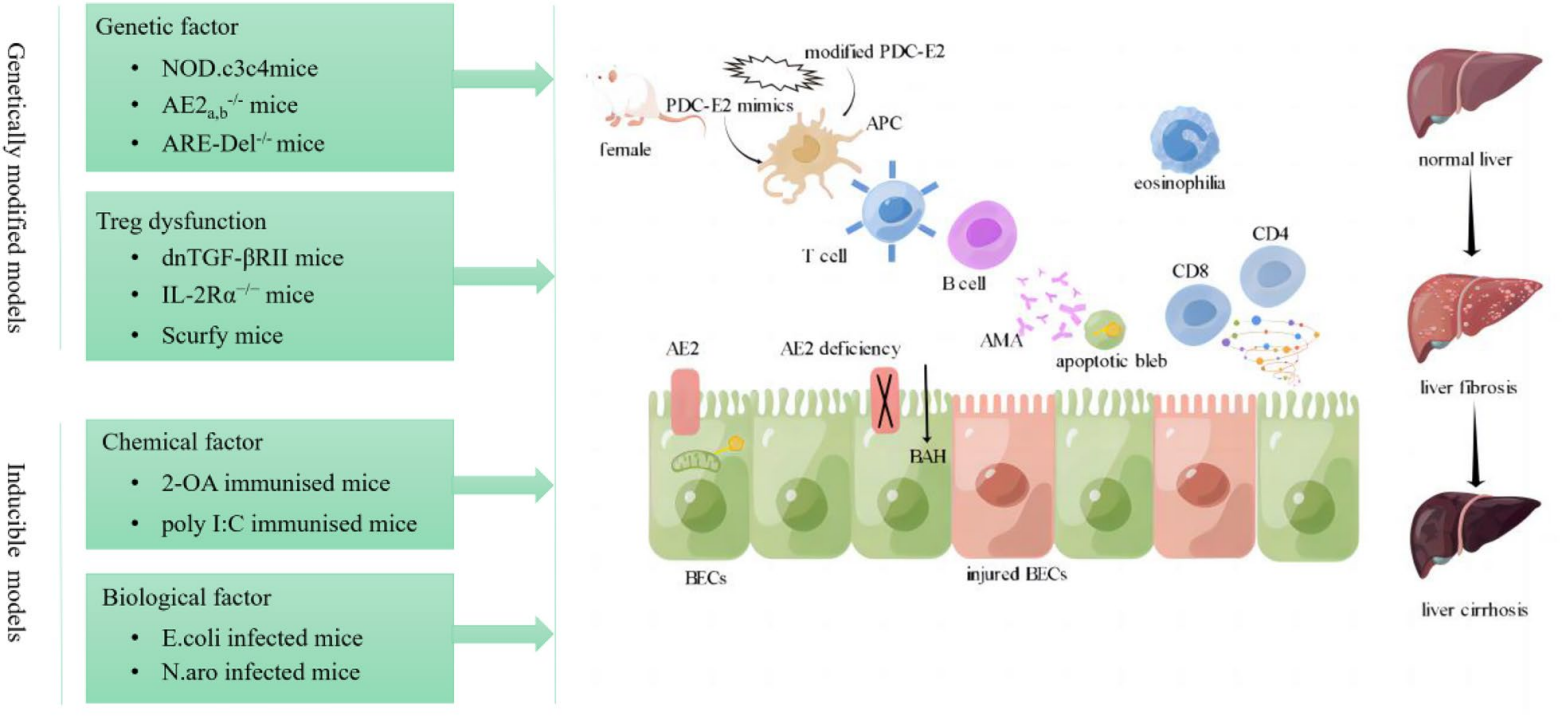
Disease characteristics in genetically modified and inducible animal models of PBC. Establishment of genetically modified models is based on genetic factors or Treg dysfunction. Chemical and infectious agents can also induce PBC development in mice. Pyruvate dehydrogenase complex (PDC)-E2 mimics or modified PDC-E2 can induce a loss of autoantigen tolerance and multilineage immune dysregulation. The main pathogenic mechanisms of PBC are between bile acid (BAH)-induced epithelial damage and autoimmune attack of bile duct epithelial cells (BECs), eventually progressing to liver fibrosis and cirrhosis

Animal models are important for research on early events and pathogenesis of PBC [15]. Various animal models have been developed, including genetically modified, chemically inducible, biologically inducible, and protein-immunised models. However, no single animal model completely simulates the clinical processes and pathogenetic mechanisms of PBC. These drawbacks restrict research and weaken links to clinical practice. Herein, we focus on the immune mechanisms and molecular targets of animal models, highlighting the shortcomings that require further optimisation. We also summarise and compare the differences in mouse strain, experimental period, modelling principle, modelling methods, serological, and histological characteristics among genetically modified mouse models, inducible mouse models, and protein-immunised mouse models (Table 1). We further summarise the advantages and disadvantages of genetically modified models of PBC (Fig. 1), and the disease characteristics in spontaneous and inducible animal models of PBC are also shown (Fig. 2).

**Table 1:** Comparison of the characteristics of animal models of primary biliary cholangitis (PBC)

Characteristics	dnTGF-βRII mice	NOD.c3c4 mice	AE2_a,b_^−/−^ mice	ARE-Del^−/−^ mice	IL-2Rα^−/−^ mice	Scurfy mice	2-OA immunised mice	*E. coli* infected mice	BDP immunised mice
Mouse strain	C57BL/6	NOD	FVB/N	C57BL/6	C57BL/6	C57BL/6	C57BL/6	NOD.B6-Idd10/Idd18	C57BL/6
Modelling principle	Abrogation of TGF-β signalling in T cells	Acquisition of insulin-dependent diabetes resistance	Dysregulation of intracellular pH in BEC and disruption of the "biliary bicarbonate umbrella"	Long-term and chronic IFN-γ overexpression	Treg dysfunction	Defective Treg function	Mimic of the lipoic acid-lysine located in the PDC-E2 domain	Cross-recognition induced by the PDC-E2 epitope	Breakdown of immune tolerance
Trigger	Transgenic	Transgenic	Transgenic	Transgenic	Transgenic	Transgenic	2-OA	*E. coli*	BDP
Experimental period	4–40 weeks	9–30 weeks	3–15 months	8–40 weeks	4–28 weeks	3–4 weeks	4–24 weeks after immunisation	4–26 weeks after infection	1 week after immunisation
Serological features									
ALP	——	——	+ ~ + +	——	——	——	——	——	——
ANA	100%	80 ~ 90%	——	——	80%	——	——	——	——
AMA	100%	50 ~ 60%	80%	100%	100%	100%	100%	100%	100%
Immunoglobulin	IgM, IgA, IgG	IgM,IgG	IgM,IgG	IgM,IgG	IgA, IgG	IgM, IgA, IgG	IgM, IgA, IgG	——	——
Histological features									
Granuloma	——	+	——	+	——	——	+	——	——
Eosinophilia	——	+	+	——	——	+	——	——	——
Lymphocytic inflammation	+ + +	+ + +	+ ~ + + +	+ + +	+ + +	+ + ~ + + +	+ ~ + +	+	+
Bile duct destruction	+ +	+	+ ~ + + +	+ ~ + +	+ ~ + +	+ ~ + +	+ ~ + +	+	——
Liver fibrosis	——	~ +	~ +	+	——	——	——	——	——

There are currently mouse models that have been studied, including genetically modified models, inducible models (2-OA immunised mice and *E. coli*-infected mice), and protein-immunised models (BDP immunised mice). Six genetically modified mouse models have been developed (dnTGF-βRII, IL-2Rα^−/−^, NOD.c3c4, AE2_a,b_^−/−^, ARE-Del^−/−^, and Scurfy mice). We summarised and compared the differences in mouse strain, experimental period, modelling principle, trigger, serological, and histological features among them

### Genetically modified models

#### Dominant-negative transforming growth factor (TGF)-β receptor II mice

The regulatory activity of circulating Tregs is dependent on TGF-β. A defect in T-cell TGF-β signalling is associated with down-regulation of T cell microRNA expression, thereby inducing cytotoxic T cell proliferative capacity and function [16, 17]. Gorelik et al. first developed dominant-negative TGF-β receptor type II (dnTGF-βRII) transgenic mice on a C57BL/6 background by overexpressing a dominant-negative form of TGF-βRII with the CD4 promoter, which lacks a CD8 silencer [18]. Oertelt et al. first reported that these mice spontaneously develop the key characteristics of PBC at 4–5 weeks, and the experimental period can be up to 40 weeks. The characteristics include 100% AMA-positivity and portal lymphocyte infiltration dominated by CD4^+^ and CD8^+^T cells, bile duct destruction, and Th1 cytokine profiles such as IFN-γ and TNF-α [19].

PBC development involves multiple immune disorders. CD8^+^T cells are key to pathogenesis in dnTGF-βRII mice. KLRG1 + lymphocytes exhibit strong cytotoxicity and positively correlate with disease severity [20]. The proportion of terminally differentiated hepatic (KLRG1 +) CD8^+^T cells is elevated in dnTGF-βRII mice [21]. Different proteins involved in chemokine signalling, focal adhesion, T cell receptors, and NK cell-mediated cytotoxicity pathways are expressed by hepatic CD8^+^T cells [22]. Among these cells, terminally differentiated CD8ααT cells exhibit a higher cytokine-producing capacity and cytotoxicity, whereas the terminally differentiated CD8αβT cells retain a proliferative profile [23]. CXC chemokine receptor 3(CXCR3) is mainly expressed on T cells. *CXCR3*-knockout dnTGF-βRII mice develop aggravated PBC and show increased frequency of KLRG1 + terminally differentiated effector memory T cells [24]. Interestingly, restoration of CD4^+^T cells can ameliorate PBC, even in the presence of pathogenetic CD8^+^T cells [25]. Hepatic NK cells differ from conventional NK cells[26]. Effector B cells generate high AMA titres [27], but the use of anti-CD20/CD79 to deplete B cells worsens PBC [28]. Thus, regulatory B cells (Bregs) play a critical role in the disease. Indeed, anti-drug antibodies that emerge in the late stages of the disease appear to be responsible for the incomplete depletion of hCD20^+^ B cells [29].

Although PBC targets cholangiocytes, the cells are safeguarded against damage by the ‘bicarbonate umbrella’. Twelve-week-old dnTGF-βRII mice exhibit elevated secretin/secretin receptor (Sct/SR) axis activation and Sct secretion. These pathways mediate biliary proliferation or senescence, as well as liver fibrosis [30]. In late disease-stage mice (32 weeks), a loss of Sct/SR signalling leads to a malfunctioning ‘bicarbonate umbrella’ [31]. Furthermore, both gut microbiota richness and diversity decrease in dnTGF-βRII mice. Translocation of gut microbiota exacerbates disease. TLR2-deficient dnTGF-βII mice exhibit down-regulated intestinal barrier function and severe cholangitis, both reversible with antibiotic treatment [32]. Antibiotics also abolish distinctions between female and male mice in terms of hepatic inflammation, suggesting that gut microbiota may drive sex differences in disease [33]. Autoimmune regulator (Aire) also plays a role in immune tolerance. dnTGFβRII Aire^−/−^ mice exhibit the major histological and serological features of autoimmune hepatitis (AIH)-PBC overlap syndrome, providing new ideas on the pathogenic mechanism of AIH-PBC [34]. dnTGF-βRII mice can also express core genes found in high-risk PBC patients, supporting the application of the mice to the research of PBC pathogenesis [35].

In recent years, dnTGF-βRII mice have been increasingly utilised in PBC studies. Based on the above studies, it can be inferred that dnTGF-βRII mice are essential for causal event analysis and investigation of key steps in PBC pathogenesis. While dnTGF-βRII mice show serological and histological features similar to those of PBC, they lack female predominance, eosinophilic infiltration, and granuloma formation. Additionally, dnTGF-βRII mice present with inflammatory bowel disease, which is rare in human PBC.

#### IL-2Ra^−/−^ mice

IL-2/IL-2R regulates Treg differentiation, and defects in IL-2Rα induce Treg dysfunction [36]. Using a C57BL/6 mouse strain, Wakabayashi et al. first demonstrated that IL-2Rα^−/−^ mice developed PBC-like features at 4–28 weeks of age. Historically, these mice have exhibited portal inflammation and biliary damage dominated by CD4^+^ and CD8^+^ T cells. Serologically, the mice are 100% AMA- and 80% antinuclear antibody (ANA)-positive, without changes in IgM levels; they produce Th1 cytokines, including IFN-γ, TNF-α, IL-2, and IL-12p40 [37]. CD8^+^T cells are the major pathogenic cells responsible for PBC in these mice. Deletion of CXCR3 resultes in enhanced liver inflammation [38]. IL-12p40 negatively regulates liver inflammation in PBC. IL-12p40^−/−^IL-2Ra^−/−^mice show manifestations of severe disease [39]. A lower number of B1a cells results in defective Treg function and worsens autoimmune response in p40^−/−^IL-2Ra^−/−^mice [40]. Furthermore, in this model, up-regulated *IL-18*, *IL-21*, and *IFN-γ* expression is involved in PBC progression. However, only IFN-γ deletion relieves both AMA and liver inflammation [41]. Antibiotic treatment mitigates splenomegaly in p40^−/−^IL-2Ra^−/−^ mice, thus reducing the associated liver inflammation [42]. IL-2Rα^−/−^mice develop colitis and severe anaemia, both of which are absent in human PBC.

#### NOD.c3c4 mice

The non-obese diabetic (NOD) mouse is a commonly used animal model for human type 1 diabetes mellitus. Understanding the genetic background of NOD mice is essential for clarifying PBC development. Koarada et al. demonstrated that NOD.c3c4 mice are resistant to type I diabetes but prone to developing autoimmune biliary disease. In these mice, multiple insulin-dependent diabetes-protective alleles derived from B6 and B10 are present on chromosomes 3 and 4 [43]. Irie et al. were the first to establish a spontaneous PBC model of NOD.c3c4 mice. The mice can develop the disease at 9–10 weeks. These mice present with 50–60% AMA and 80–90% ANA. Histology shows eosinophil infiltration, granuloma formation, and lymphocyte peribiliary infiltration dominated by CD4^+^ and CD8^+^T cells [44]. Adoptive transfer experiments have shown that only CD4^+^ or CD8^+^T cells can trigger liver inflammation. Igμ^−/−^ NOD.c3c4 mice deficient in B cells exhibit attenuated hepatic inflammation [45]. Gut microbiota influences spontaneous bile duct inflammation in mice [46]. Human β retroviruses, which can be isolated from PBC, have approximately 95% nucleotide homology with mouse mammary tumour virus (MMTV) [47]. MMTV protein has been detected in damaged BECs of NOD.c3c4 mice, and MMTV infection may trigger AMA production [48]. Combined retroviral treatment to inhibit MMTV replication substantially ameliorates intrahepatic cholangitis in NOD.c3c4 mice [49, 50].

The autoimmune biliary disease of NOD.c3c4 mice differs from PBC in the site of initial autoimmune attack, occurring in the common bile duct rather than intrahepatic BECs. Older mice (30 weeks) develop common bile duct dilation and biliary polycysts, which even mask the nonsuppurative destructive cholangitis-like lesion. Some mice produce systemic lupus erythematosus (SLE)-specific anti-Smith antibodies, an uncommon feature of human PBC.

#### AE2_a,b_^−/−^ mice

Cl^−^/HCO_3_^−^ anion exchanger 2 (AE2), expressed on cholangiocytes, is involved in the maintenance of biliary intracellular pH (pHi) and ‘bicarbonate umbrella’ homeostasis. If the ‘bicarbonate umbrella’ is destroyed, toxic bile salts penetrate cholangiocytes and induce apoptosis [51]. AE2_a,b_^−/−^ mice have an FVB/N background studied at 3–15 months. Serologically, most AE2_a,b_^−/−^ mice are AMA-positive and exhibit elevated IgM and ALP. Histology shows that approximately one-third of these mice develop lymphocytic infiltration around the bile duct and mild fibrosis [52]. AE2 is also expressed in lymphocytes, and CD8^+^T cells are heavily dependent on AE2 to regulate pHi. CD8^+^T cell-deficiency in AE2 mice increases IL-2 production, promoting cellular proliferation and activation upon stimulation [53]. AE2 deficiency in young AE2_a,b_^−/−^ mice leads to intrahepatic T cell activation; however, PD-1/PD-L1 promotes T cell apoptosis, thereby preventing autoimmune damage. DNA methylation of liver CD8^+^T cells in older mice weakens the effect of PD1/PD-L1 [54].

In contrast to human patients with PBC, AE2_a,b_^−/−^ mice exhibit delayed disease onset and slow progression. Furthermore, reproduction of these animals is difficult, restricting their application.

#### ARE-Del^−/−^ mice

Th1 cytokines are involved in PBC progression. IFN-γ levels are elevated in the serum of patients with PBC. Hodge et al. generated a PBC mouse model on a C57BL/6 background by deleting the 3′-untranslated region of the uridylate-rich adenylate element of IFN, resulting in long-term and chronic IFN-γ overexpression. These ARE-Del^−/−^ mice exhibit serological and pathologic features of PBC at experimental periods of 8–40 weeks, but more importantly, the features are female-dominant [55]. Key pathogenic cells in ARE-Del^−/−^ mice are CD4^+^T cells, indicating that IFN-γ is important during the early stages of PBC development. Downstream signalling molecules of IFNs are JAK1 and JAK2. The United States Food and Drug Administration-approved JAK1 inhibitor ruxolitinib improves liver pathology and AMA levels [56]. Type I IFN signalling may also be a contributing factor in the mouse model of PBC. Indeed, the disease is substantially ameliorated in ARE-Del^−/−^IFN-aR1^−/−^ mice, and sex differences are diminished [57]. Furthermore, failure to treat human PBC with rituximab is associated with elevated levels of B-cell activating factor (BAFF). When treated with anti-BAFF and anti-CD20 monoclonal antibodies, the disease is alleviated in ARE-Del^−/−^ mice [58].

These features indicate that ARE-Del^−/−^ mice can be used to explore the underlying mechanisms of sex differences in PBC. However, ARE-Del^−/−^ mice produce anti-DNA antibodies while displaying typical serum and cellular abnormalities in systemic lupus erythematosus.

### Chemically inducible models

#### 2-Octynoic acid (2-OA)-immunised mice

2-OA is a synthetic chemical mimic of the lipoic acid-lysine located in the PDC-E2 domain. Mice from the C57BL/6 background immunised with 2-OA exhibit the majority of PBC characteristics, including 100% AMA, elevated IFN-γ and TNF-α levels, portal inflammation, granuloma formation, and biliary damage dominated by CD4^+^T and CD8^+^T cells [59]. 8–9-week-old mice can be studied at 4–24 weeks after immunisation.

Th1 cells and IFN-γ are the major contributors during early-stage PBC development. In this stage, 2-OA-conjugated ovalbumin immunised mice injected with adeno-associated virus (AAV)-IFN-γ exhibit enhanced liver inflammation. However, as the disease progresses, an increase in the IL-30 level alleviates chronic inflammation [60]. Notably, another study showed that IL-30 treatment inhibits both CD4^+^T cells and Tregs [61]. Transplantation of human umbilical cord-derived mesenchymal stem cells into 2-OA-bovine serum albumin (BSA) mice considerably ameliorates hepatic inflammation by dampening the Th1/Th17 response [62]. Moreover, the absence of Th17 cells inhibits hepatic accumulation of IFN-γ-producing cells, but IL-23/Th17 cells promote Th1-mediated immunopathology [63]. Th2 cells and their secreted cytokines are also implicated in PBC pathogenesis. Endogenous IL-10 is one such immunosuppressive anti-inflammatory cytokine. Administration of exogenous AAV-IL-10 promotes lymphocyte infiltration and collagen deposition [64]. The injection of the IL-10 member AAV-IL-22 prevents clinical autoimmune cholangitis and substantially ameliorates portal inflammation, even in mice with pre-existing clinical pathology [65]. In addition to adaptive immunity, mice with mononuclear phagocyte-restricted IL-23 deficiency exhibit lower disease severity, because fewer CD4^+^T cells are present in the liver to produce IL-17A and IL-23/IL-17 [66]. Another study showed that infiltrating Ly6C^hi^ monocytes in 2-OA-BSA mice are recruited to the portal area in the form of C–C motif chemokine receptor 2, worsening histopathological symptoms [67].

In summary, 2-OA immunised mice can be used to explore the association between environmental factors and PBC progression. However, the natural history of disease is less severe than that of human PBC.

#### Polyinosine polypeptidic acid (poly I:C)-sensitised mice

Poly I:C can mimic viral RNA and induces type 1 IFN production. High IFN expression is linked to the progression of autoimmune disease. Okada et al. generated poly I:C sensitised mice with lesions resembling PBC. The lesions appear earlier in the liver than those in spontaneous models [68]. However, these mice also exhibit extrahepatic inflammatory lesions in the salivary glands, pancreas, and kidneys. In addition to inhibiting the inflammatory response, TGF-β1 promotes fibrosis in poly I:C mice [69]. Research using these mice has revealed that nuclear factor kappa B (NF-κB) is involved in PBC pathophysiology. Activation of TLR4/MyD88/NF-κB signalling promotes the release of inflammatory cytokines, subsequently leading to apoptosis [70]. However, p65 subunit deacetylation and dephosphorylation block NF-κB activity in poly I:C mice [71].

#### 2-OA and poly I:C co-immunised mice

The introduction of poly I:C into 2-OA mice worsens autoimmune cholangitis, increasing eosinophil infiltration and evidence of fibrosis. Alkaline phosphatase and Th1 cytokine levels increase substantially, along with alanine transaminase and aspartate transaminase levels [72]. Approximately 5% of lymphocytes infiltrating the bile ducts are NK and NKT cells. Tim-3 regulates the local liver immune microenvironment through modulating CXCR3 and CXCR1 expression in NK cells of 2-OA/poly I:C co-immunised mice. Furthermore, liver Kupffer cells interact with NK cells via NKG2D/RAE-1 to induce NK cell-mediated injury in these mice [73]. The *Clostridium* metabolite p-Cresol sulphate can lower PBC-related inflammation by altering Kupffer cell polarisation [74]. Additionally, dysregulation of Treg/Th17 cells is implicated in damage to the liver microenvironment [75]. FoxP3 demethylation mediated by 5-aza-2-deoxycytidine restores Treg/Th17 balance, relieving portal infiltration and liver damage [76].

### Biologically inducible models

#### Escherichia coli-infected mice

Several studies have confirmed that a history of urinary tract infection increases the risk of PBC [77]. The causative agent of most urinary tract infections is *E. coli*. As the bacterium mimics the mechanism of cross-recognition induced by the PDC-E2 epitope in PBC [78, 79], infection decreases organ specificity. The PDC-E2-associated TCRβ repertoire features of memory T cells are nearly identical between *E. coli* and human PBC [80]. Similarly, the AMA levels peak at 4 weeks in *E. coli*-infected NOD.B6-Idd10/Idd18 mice; portal inflammation and granuloma formation are observed at 26 weeks, along with extensive biliary damage [81].

### Protein-immunised models

#### Bile duct protein(BDP)-immunised mice

Mouse models of xenobiotic and infectious agents are generated from molecular simulation or modification of PDC-E2. Ma et al. generated the classical break-tolerance model with a C57BL/6 background via immunisation with bile duct protein. After one week of bile duct protein immunity, the mice can initiate lymphocytic infiltration and elevate AMA levels. However, experiments have demonstrated that inducing autoimmune cholangitis in male mice is difficult [82].

### Other mouse models

Scurfy mice from a C57BL/6 background are deficient in normal functional Tregs because of mutations in the gene encoding the Foxp3 transcription factor. Unfortunately, most mice die at 4 weeks, limiting their application in long-term studies of PBC [83].

*Novosphingobium aromaticivorans* is a bacterium belonging to a strictly aerobic gram-negative genus with amino acid sequences highly homologous to those of PDC-E2 [84]. Compared to chemically inducible models, *N. aromaticivorans*-infected mice exhibit characteristics that are more similar to the natural history of PBC [85]. Accumulation of *N. aromaticivorans* in the liver causes chronic inflammation, accounting for the organ specificity of PBC.

The activation of inflammatory cells is controlled at both transcriptional and post-transcriptional levels by monocyte chemotactic protein-induced protein 1 (MCPIP1). Recently, Kotlinowski et al. demonstrated that the phenotype of Mcpip1^fl/fl^Alb^cre^ mice is similar to the serological and histological characteristics of PBC. Additionally, aging aggravated the disease’s progression, with the mice showing substantial cholestasis and progressive liver fibrosis [86].

### Summary

Selecting an appropriate animal model enables convenient causal analysis and further research when studying PBC as well as evaluation of the efficacy of new treatments. The characteristics of an ideal PBC animal model include female predominance, AMA production, intrahepatic bile duct destruction, lymphocytic infiltration, granuloma formation, eosinophilia, and liver fibrosis. Genetically modified models reveal the important role of genetic factors and provide ideas for studying the early events and pathogenic mechanisms of PBC, but most of them lack organ specificity or have no female dominance. In recent years, dnTGF-βRII mice have been used more often for PBC studies. dnTGF-βRII mice enable causal analysis and elucidation of the key steps in the pathogenesis of PBC, allowing for a deeper understanding of the disease. These mice can represent PBC better, but still have limitations. Chemically and biologically inducible models are suitable for exploring early pathogenic processes caused by environmental factors. Protein-immunised models provide a basis for bile duct antigenic pathogenesis. These inducible models have a short modelling time, but the experimental costs and conditions are stringent. They rarely progress to advanced stages. However, improvement options for the shortcomings of the animal models still need to be further explored, and a new mouse model that completely mimics human PBC is yet to be developed.

### Perspectives and challenges

Genetic susceptibility does not fully explain the risk of PBC, indicating that the involvement of environmental factors. Despite the clear involvement of the immune system, PBC treatments are aimed at regulating bile acid metabolism rather than the immune effector pathway. Therefore, the immune mechanisms require further elucidation. Although several animal models have been studied, no single animal model can completely mimic the complex aetiologies and mechanisms. In particular, because patients are asymptomatic during the early stages of PBC, studying early pathogenesis is extremely challenging in humans. This difficulty should motivate the construction and optimisation of PBC animal models, a critical element to advance research on early-stage pathogenesis of PBC and the molecular targets for therapeutic intervention. We can attempt to discover novel susceptibility genes within PBC patient pedigrees and then construct a genetically modified mouse model based on the gene, which can help to effectively explore the relationship between susceptibility genes and the pathogenesis of PBC. Using this approach, our team is currently studying a new animal model of PBC.

## Data Availability

Not applicable.

## Abbreviations

2-OA: 2-Octanoic acid
AAV: Adeno-associated virus
AE2: Anion exchanger 2
ALP: Alkaline phosphatase
AMA: Antimitochondrial antibody
ANA: Antinuclear antibody
BAFF: B-cell activating factor
BDP: Bile duct protein
BECs: Bile duct epithelial cells
BSA: Bovine serum albumin
dnTGF-βRII: Dominant-negative TGF-β receptor type II
Ig: M immunoglobulin
PDC-E2: E2 subunit of pyruvate dehydrogenase complex
IFN: Interferon
pHi: Intracellular pH
MCPIP1: Monocyte chemotactic protein-induced protein 1
MMTV: Mouse mammary tumour virus
NK: Natural killer
NOD: Non-obese diabetic
NF-κB: Nuclear factor kappa B
poly I:C: Polyinosine polypeptidic acid
PBC: Primary biliary cholangitis
Breg: Regulatory B cell
Treg: Regulatory T cell
Sct/SR: Secretin/secretin receptor
TGF-β: Transforming growth factor
TNF-α: Tumour necrosis factor

## References

[1] Shimoda S, Tanaka A (2016). It is time to change primary biliary cirrhosis (PBC): new nomenclature from "cirrhosis" to "cholangitis", and upcoming treatment based on unveiling pathology. Hepatol Res.

[2] Trivedi PJ, Hirschfield GM (2021). Recent advances in clinical practice: epidemiology of autoimmune liver diseases. Gut.

[3] Zeng N, Duan W, Chen S, Wu S, Ma H, Ou X (2019). Epidemiology and clinical course of primary biliary cholangitis in the Asia-Pacific region: a systematic review and meta-analysis. Hepatol Int.

[4] Gerussi A, Cristoferi L, Carbone M, Asselta R, Invernizzi P (2018). The immunobiology of female predominance in primary biliary cholangitis. J Autoimmun.

[5] Ellinghaus D (2022). How genetic risk contributes to autoimmune liver disease. Semin Immunopathol.

[6] Matsumoto K, Ohfuji S, Abe M, Komori A, Takahashi A, Fujii H (2022). Environmental factors, medical and family history, and comorbidities associated with primary biliary cholangitis in Japan: a multicenter case-control study. J Gastroenterol.

[7] Ma WT, Chen DK (2019). Immunological abnormalities in patients with primary biliary cholangitis. Clin Sci (Lond).

[8] Chen Z, Liu S, He C, Sun J, Wang L, Chen H (2021). CXCL12-CXCR4-mediated chemotaxis supports accumulation of mucosal-associated invariant T cells into the liver of patients with PBC. Front Immunol.

[9] Schrumpf E, Tan C, Karlsen TH, Sponheim J, Björkström NK, Sundnes O (2015). The biliary epithelium presents antigens to and activates natural killer T cells. Hepatology.

[10] Li X, Li Y, Xiao J, Wang H, Guo Y, Mao X (2023). Unique DUOX2(+)ACE2(+) small cholangiocytes are pathogenic targets for primary biliary cholangitis. Nat Commun.

[11] Younossi ZM, Bernstein D, Shiffman ML, Kwo P, Kim WR, Kowdley KV (2019). diagnosis and management of primary biliary cholangitis. Am J Gastroenterol.

[12] Laschtowitz A, de Veer RC, Van der Meer AJ, Schramm C (2020). Diagnosis and treatment of primary biliary cholangitis. United European Gastroenterol J.

[13] Liu C-H, Bowlus CL (2022). Treatment of Primary Biliary Cholangitis: First-Line and Second-Line Therapies. Clin Liver Dis.

[14] Terziroli Beretta-Piccoli B, Mieli-Vergani G, Vergani D, Vierling JM, Adams D, Alpini G (2019). The challenges of primary biliary cholangitis: What is new and what needs to be done. J Autoimmun.

[15] Liu S-P, Bian Z-H, Zhao Z-B, Wang J, Zhang W, Leung PSC (2020). Animal models of autoimmune liver diseases: a comprehensive review. Clin Rev Allergy Immunol.

[16] Itoh A, Adams D, Huang W, Wu Y, Kachapati K, Bednar KJ (2021). Enoxacin up-regulates MicroRNA biogenesis and down-regulates cytotoxic CD8 T-cell function in autoimmune cholangitis. Hepatology.

[17] Kawata K, Yang G-X, Ando Y, Tanaka H, Zhang W, Kobayashi Y (2013). Clonality, activated antigen-specific CD8(+) T cells, and development of autoimmune cholangitis in dnTGFβRII mice. Hepatology.

[18] Gorelik L, Flavell RA (2000). Abrogation of TGFbeta signaling in T cells leads to spontaneous T cell differentiation and autoimmune disease. Immunity.

[19] Oertelt S, Lian Z-X, Cheng C-M, Chuang Y-H, Padgett KA, He X-S (2006). Anti-mitochondrial antibodies and primary biliary cirrhosis in TGF-beta receptor II dominant-negative mice. J Immunol.

[20] Li Y, Li B, You Z, Zhang J, Wei Y, Li Y (2019). Cytotoxic KLRG1 expressing lymphocytes invade portal tracts in primary biliary cholangitis. J Autoimmun.

[21] Huang W, Kachapati K, Adams D, Wu Y, Leung PSC, Yang G-X (2014). Murine autoimmune cholangitis requires two hits: cytotoxic KLRG1(+) CD8 effector cells and defective T regulatory cells. J Autoimmun.

[22] Zhang W, Zhang R, Zhang J, Sun Y, Leung PS, Yang G-X (2018). Proteomic analysis reveals distinctive protein profiles involved in CD8+ T cell-mediated murine autoimmune cholangitis. Cell Mol Immunol.

[23] Han Y, Bian Z-H, Yang S-Y, Wang C-B, Li L, Yang Y-Q (2022). Single-cell characterization of hepatic CD8 T cells in a murine model of primary biliary cholangitis. Front Immunol.

[24] Ma H-D, Ma W-T, Liu Q-Z, Zhao Z-B, Liu M-Z-Y, Tsuneyama K (2017). Chemokine receptor CXCR3 deficiency exacerbates murine autoimmune cholangitis by promoting pathogenic CD8 T cell activation. J Autoimmun.

[25] Yang J-B, Wang Y-H, Yang W, Lu F-T, Ma H-D, Zhao Z-B (2016). Successful treatment of murine autoimmune cholangitis by parabiosis: implications for hematopoietic therapy. J Autoimmun.

[26] Zhao ZB, Lu FT, Ma HD, Wang YH, Yang W, Long J (2020). Liver-resident NK cells suppress autoimmune cholangitis and limit the proliferation of CD4(+) T cells. Cell Mol Immunol.

[27] Leung PSC, Choi J, Yang G, Woo E, Kenny TP, Gershwin ME (2016). A contemporary perspective on the molecular characteristics of mitochondrial autoantigens and diagnosis in primary biliary cholangitis. Expert Rev Mol Diagn.

[28] Dhirapong A, Lleo A, Yang G-X, Tsuneyama K, Dunn R, Kehry M (2011). B cell depletion therapy exacerbates murine primary biliary cirrhosis. Hepatology.

[29] Moritoki Y, Tsuneyama K, Nakamura Y, Kikuchi K, Shiota A, Ohsugi Y (2018). Anti-drug antibodies against a novel humanized anti-CD20 antibody impair its therapeutic effect on primary biliary cholangitis in human CD20- and FcgammaR-expressing mice. Front Immunol.

[30] Kennedy L, Francis H, Invernizzi P, Venter J, Wu N, Carbone M (2019). Secretin/secretin receptor signaling mediates biliary damage and liver fibrosis in early-stage primary biliary cholangitis. FASEB J.

[31] Kennedy L, Carpino G, Owen T, Ceci L, Kundu D, Meadows V (2023). Secretin alleviates biliary and liver injury during late-stage primary biliary cholangitis via restoration of secretory processes. J Hepatol.

[32] Ma HD, Zhao ZB, Ma WT, Liu QZ, Gao CY, Li L (2018). Gut microbiota translocation promotes autoimmune cholangitis. J Autoimmun.

[33] Huang MX, Yang SY, Luo PY, Long J, Liu QZ, Wang J (2021). Gut microbiota contributes to sexual dimorphism in murine autoimmune cholangitis. J Leukoc Biol.

[34] Long J, Yang SY, Huang MX, Luo PY, Li L, Tsuneyama K (2023). Spontaneous development of an autoimmune hepatitis - primary biliary cholangitis overlap syndrome in dnTGFβRII Aire(-/-) mice. J Pathol.

[35] Tian S, Hu Y, Zhang M, Wang K, Guo G, Li B (2023). Integrative bioinformatics analysis and experimental validation of key biomarkers for risk stratification in primary biliary cholangitis. Arthritis Res Ther.

[36] Li Y, Li X, Geng X, Zhao H (2022). The IL-2A receptor pathway and its role in lymphocyte differentiation and function. Cytokine Growth Factor Rev.

[37] Wakabayashi K, Lian Z-X, Moritoki Y, Lan RY, Tsuneyama K, Chuang Y-H (2006). IL-2 receptor alpha(-/-) mice and the development of primary biliary cirrhosis. Hepatology.

[38] Liu Q-Z, Ma W-T, Yang J-B, Zhao Z-B, Yan K, Yao Y (2018). The CXC chemokine receptor 3 inhibits autoimmune cholangitis CD8 T cells but promotes colitis CD4 T cells. Front Immunol.

[39] Yao Y, Yang W, Yang Y-Q, Ma H-D, Lu F-T, Li L (2014). Distinct from its canonical effects, deletion of IL-12p40 induces cholangitis and fibrosis in interleukin-2Rα(-/-) mice. J Autoimmun.

[40] Yang Y-Q, Yang W, Yao Y, Ma H-D, Wang Y-H, Li L (2016). Dysregulation of peritoneal cavity B1a cells and murine primary biliary cholangitis. Oncotarget.

[41] Xu YF, Yao Y, Ma M, Yang SH, Jiang P, Wang J (2022). The Proinflammatory Cytokines IL-18, IL-21, and IFN-gamma differentially regulate liver inflammation and anti-mitochondrial antibody level in a murine model of primary biliary cholangitis. J Immunol Res.

[42] Wang C-B, Wang Y, Yao Y, Wang J-J, Tsuneyama K, Yang Q (2022). The gut microbiome contributes to splenomegaly and tissue inflammation in a murine model of primary biliary cholangitis. Ann Transl Med.

[43] Koarada S, Wu Y, Fertig N, Sass DA, Nalesnik M, Todd JA (2004). Genetic control of autoimmunity: protection from diabetes, but spontaneous autoimmune biliary disease in a nonobese diabetic congenic strain. J Immunol.

[44] Irie J, Wu Y, Wicker LS, Rainbow D, Nalesnik MA, Hirsch R (2006). NOD.c3c4 congenic mice develop autoimmune biliary disease that serologically and pathogenetically models human primary biliary cirrhosis. J Exp Med.

[45] Moritoki Y, Tsuda M, Tsuneyama K, Zhang W, Yoshida K, Lian Z-X (2011). B cells promote hepatic inflammation, biliary cyst formation, and salivary gland inflammation in the NOD.c3c4 model of autoimmune cholangitis. Cell Immunol.

[46] Schrumpf E, Kummen M, Valestrand L, Greiner TU, Holm K, Arulampalam V (2017). The gut microbiota contributes to a mouse model of spontaneous bile duct inflammation. J Hepatol.

[47] Goubran M, Wang W, Indik S, Faschinger A, Wasilenko ST, Bintner J (2022). Isolation of a human betaretrovirus from patients with primary biliary cholangitis. Viruses.

[48] Zhang G, Chen M, Graham D, Subsin B, McDougall C, Gilady S (2011). Mouse mammary tumor virus in anti-mitochondrial antibody producing mouse models. J Hepatol.

[49] Sharon D, Chen M, Zhang G, Girgis S, Sis B, Graham D (2015). Impact of combination antiretroviral therapy in the NOD.c3c4 mouse model of autoimmune biliary disease. Liver Int.

[50] Syed H, Penner T, Mason AL (2022). Linking Human Betaretrovirus with autoimmunity and liver disease in patients with primary biliary cholangitis. Viruses.

[51] Arenas F, Hervías I, Sáez E, Melero S, Prieto J, Parés A (2019). Promoter hypermethylation of the *AE2/SLC4A2* gene in PBC. JHEP Rep.

[52] Salas JT, Banales JM, Sarvide S, Recalde S, Ferrer A, Uriarte I (2008). Ae2a, b-deficient mice develop antimitochondrial antibodies and other features resembling primary biliary cirrhosis. Gastroenterology.

[53] Concepcion AR, Salas JT, Sarvide S, Sáez E, Ferrer A, López M (2014). Anion exchanger 2 is critical for CD8(+) T cells to maintain pHi homeostasis and modulate immune responses. Eur J Immunol.

[54] Concepcion AR, Salas JT, Sáez E, Sarvide S, Ferrer A, Portu A (2015). CD8+ T cells undergo activation and programmed death-1 repression in the liver of aged Ae2a, b-/- mice favoring autoimmune cholangitis. Oncotarget.

[55] Bae HR, Leung PSC, Tsuneyama K, Valencia JC, Hodge DL, Kim S (2016). Chronic expression of interferon-gamma leads to murine autoimmune cholangitis with a female predominance. Hepatology.

[56] Shao T, Leung PSC, Zhang W, Tsuneyama K, Ridgway WM, Young HA (2022). Treatment with a JAK1/2 inhibitor ameliorates murine autoimmune cholangitis induced by IFN overexpression. Cell Mol Immunol.

[57] Bae HR, Hodge DL, Yang G-X, Leung PSC, Chodisetti SB, Valencia JC (2018). The interplay of type I and type II interferons in murine autoimmune cholangitis as a basis for sex-biased autoimmunity. Hepatology.

[58] Zhang W, Shao T, Leung PSC, Tsuneyama K, Heuer L, Young HA (2022). Dual B-cell targeting therapy ameliorates autoimmune cholangitis. J Autoimmun.

[59] Wakabayashi K, Lian Z-X, Leung PSC, Moritoki Y, Tsuneyama K, Kurth MJ (2008). Loss of tolerance in C57BL/6 mice to the autoantigen E2 subunit of pyruvate dehydrogenase by a xenobiotic with ensuing biliary ductular disease. Hepatology.

[60] Syu B-J, Loh C-E, Hsueh Y-H, Gershwin ME, Chuang Y-H (2016). Dual Roles of IFN-γ and IL-4 in the natural history of murine autoimmune cholangitis: IL-30 and implications for precision medicine. Sci Rep.

[61] Chen H-W, Lin C-I, Chuang Y-H (2021). Interleukin-30 suppresses Not Only CD4+ T cells but also regulatory T Cells in murine primary biliary cholangitis. Biomedicines.

[62] Fan J, Tang X, Wang Q, Zhang Z, Wu S, Li W (2018). Mesenchymal stem cells alleviate experimental autoimmune cholangitis through immunosuppression and cytoprotective function mediated by galectin-9. Stem Cell Res Ther.

[63] Kawata K, Tsuda M, Yang G-X, Zhang W, Tanaka H, Tsuneyama K (2013). Identification of potential cytokine pathways for therapeutic intervention in murine primary biliary cirrhosis. PLoS ONE.

[64] Hsueh YH, Chen HW, Syu BJ, Lin CI, Leung PSC, Gershwin ME (2018). Endogenous IL-10 maintains immune tolerance but IL-10 gene transfer exacerbates autoimmune cholangitis. J Autoimmun.

[65] Hsueh Y-H, Chang Y-N, Loh C-E, Gershwin ME, Chuang Y-H (2016). AAV-IL-22 modifies liver chemokine activity and ameliorates portal inflammation in murine autoimmune cholangitis. J Autoimmun.

[66] Reuveni D, Brezis MR, Brazowski E, Vinestock P, Leung PSC, Thakker P (2021). Interleukin 23 produced by hepatic monocyte-derived macrophages is essential for the development of murine primary biliary cholangitis. Front Immunol.

[67] Reuveni D, Gore Y, Leung PSC, Lichter Y, Moshkovits I, Kaminitz A (1852). The critical role of chemokine (C-C Motif) receptor 2-positive monocytes in autoimmune cholangitis. Front Immunol.

[68] Okada C, Akbar SMF, Horiike N, Onji M (2005). Early development of primary biliary cirrhosis in female C57BL/6 mice because of poly I: C administration. Liver Int.

[69] Liu B, Zhang X, Zhang F-C, Zong J-B, Zhang W, Zhao Y (2013). Aberrant TGF-β1 signaling contributes to the development of primary biliary cirrhosis in murine model. World J Gastroenterol.

[70] Yu Y, Li MP, Xu B, Fan F, Lu SF, Pan M (2019). A study of regulatory effects of TLR4 and NF-kappaB on primary biliary cholangitis. Eur Rev Med Pharmacol Sci.

[71] Li Y, Xi Y, Tao G, Xu G, Yang Z, Fu X (2020). Sirtuin 1 activation alleviates primary biliary cholangitis via the blocking of the NF-kappaB signaling pathway. Int Immunopharmacol.

[72] Xu J, Fu H, Yang Y, Yu H, Ai X, Lei Y (2021). Modulation of CXCR1 and CXCR3 expression on NK cells via Tim-3 in a murine model of primary biliary cholangitis. Mol Immunol.

[73] Fu HY, Bao WM, Yang CX, Lai WJ, Xu JM, Yu HY (2020). Kupffer cells regulate natural killer cells via the NK group 2, Member D (NKG2D)/retinoic acid early inducible-1 (RAE-1) interaction and cytokines in a primary biliary cholangitis mouse model. Med Sci Monit.

[74] Fu H-Y, Xu J-M, Ai X, Dang F-T, Tan X, Yu H-Y (2022). The clostridium metabolite P-cresol sulfate relieves inflammation of primary biliary cholangitis by regulating kupffer cells. Cells.

[75] Yang C-Y, Ma X, Tsuneyama K, Huang S, Takahashi T, Chalasani NP (2014). IL-12/Th1 and IL-23/Th17 biliary microenvironment in primary biliary cirrhosis: implications for therapy. Hepatology.

[76] Jiang T, Zhang H-W, Wen Y-P, Yin Y-S, Yang L-H, Yang J (2021). 5-Aza-2-deoxycytidine alleviates the progression of primary biliary cholangitis by suppressing the FoxP3 methylation and promoting the Treg/Th17 balance. Int Immunopharmacol.

[77] Corpechot C, Chrétien Y, Chazouillères O, Poupon R (2010). Demographic, lifestyle, medical and familial factors associated with primary biliary cirrhosis. J Hepatol.

[78] Tanaka A, Leung PSC, Gershwin ME (2019). Pathogen infections and primary biliary cholangitis. Clin Exp Immunol.

[79] Yang Y, Choi J, Chen Y, Invernizzi P, Yang G, Zhang W (2022). *E. coli* and the etiology of human PBC: Antimitochondrial antibodies and spreading determinants. Hepatology.

[80] Hou X, Yang Y, Chen J, Jia H, Zeng P, Lv L (2019). TCRβ repertoire of memory T cell reveals potential role for Escherichia coli in the pathogenesis of primary biliary cholangitis. Liver Int.

[81] Wang JJ, Yang GX, Zhang WC, Lu L, Tsuneyama K, Kronenberg M (2014). *Escherichia coli* infection induces autoimmune cholangitis and anti-mitochondrial antibodies in non-obese diabetic (NOD).B6 (Idd10/Idd18) mice. Clin Exp Immunol.

[82] Ma WT, Liu QZ, Yang JB, Yang YQ, Zhao ZB, Ma HD (2017). A mouse model of autoimmune cholangitis via syngeneic bile duct protein immunization. Sci Rep.

[83] Zhang W, Sharma R, Ju S-T, He X-S, Tao Y, Tsuneyama K (2009). Deficiency in regulatory T cells results in development of antimitochondrial antibodies and autoimmune cholangitis. Hepatology.

[84] Padgett KA, Selmi C, Kenny TP, Leung PSC, Balkwill DL, Ansari AA (2005). Phylogenetic and immunological definition of four lipoylated proteins from Novosphingobium aromaticivorans, implications for primary biliary cirrhosis. J Autoimmun.

[85] Selmi C, Balkwill DL, Invernizzi P, Ansari AA, Coppel RL, Podda M (2003). Patients with primary biliary cirrhosis react against a ubiquitous xenobiotic-metabolizing bacterium. Hepatology.

[86] Kotlinowski J, Hutsch T, Czyzynska-Cichon I, Wadowska M, Pydyn N, Jasztal A (2021). Deletion of Mcpip1 in Mcpip1(fl/fl)Alb(Cre) mice recapitulates the phenotype of human primary biliary cholangitis. Biochim Biophys Acta Mol Basis Dis.

